# Photon-counting computed tomography for paediatric congenital heart defects yields images of high diagnostic quality with low radiation doses at both 70 kV and 90 kV

**DOI:** 10.1007/s00247-024-05939-z

**Published:** 2024-05-03

**Authors:** Fredrik Stålhammar, Marie-Louise Aurumskjöld, Sofie Meyer, Marie Wiklund, Pär Wingren, Petru Liuba, Erik Hedström

**Affiliations:** 1 Diagnostic Radiology, Department of Clinical Sciences Lund, Lund University, Lund, Sweden; 2 Department of Radiology, Skåne University Hospital, S-22185 Lund, Sweden; 3 Medical Radiation Physics, Department of Clinical Sciences Malmö, Lund University, Lund, Sweden; 4 Radiation Physics, Department of Haematology, Oncology and Radiation Physics, Skåne University Hospital, Lund, Sweden; 5 Paediatric Cardiology, Department of Clinical Sciences Lund, Lund University, Lund, Sweden; 6 Department of Paediatric Cardiology, Skåne University Hospital, Lund, Sweden; 7 Clinical Physiology, Department of Clinical Sciences Lund, Lund University, Lund, Sweden; 8 Department of Clinical Physiology, Skåne University Hospital, Lund, Sweden

**Keywords:** Diagnosis, Heart defects, congenital, Paediatrics, Photon-counting computed tomography, Radiation dosage

## Abstract

**Background:**

Photon-counting computed tomography (PCCT) is a new clinical method that may show better diagnostic quality at lower radiation doses than conventional CT.

**Objective:**

To investigate the diagnostic quality and radiation dose of paediatric cardiovascular PCCT for diagnosis of congenital heart defects at 70 kV and 90 kV.

**Materials and methods:**

This retrospective assessment included clinical non-gated paediatric PCCT examinations for assessment of congenital heart defects. Radiation doses were recorded, and overall and specific diagnostic quality (1–4) were scored by four paediatric radiologists. Agreement, differences, and trends were assessed by percent rater agreement, intraclass correlation, Mann–Whitney tests, and Jonckheere-Terpstra tests.

**Results:**

Seventy children with congenital heart defects were examined at 70 kV (*n* = 35; age 2 days–16 years; 63% boys) or 90 kV (*n* = 35; age 2 days–17 years; 51% boys). All observers gave a median score of 4 (high diagnostic quality) for both 70 kV and 90 kV, with no difference in median values between tube voltages (all *P* > 0.06). Agreement for overall scores was 66–94% for 70 kV and 60–77% for 90 kV. Agreement for specific scores was 80–97% for 70 kV and 83–89% for 90 kV. Size-dependent dose estimate was 0.68 mGy (0.25–2.02 mGy) for 70 kV and 1.10 mGy (0.58–2.71 mGy; *P* < 0.001) for 90 kV. Effective dose was 0.30 mSv (0.15–0.82 mSv) for 70 kV and 0.39 mSv (0.22–1.51 mSv; *P* = 0.01) for 90 kV.

**Conclusion:**

Paediatric cardiovascular PCCT yields images for congenital heart defects of high diagnostic quality with low radiation dose at both 70 kV and 90 kV.

## Introduction

Computed tomography (CT) is used in paediatric patients with congenital heart defects for diagnosis, preoperative planning, and postoperative evaluation [1–3]. CT is widely available and fast. It can often be used without sedation or with feed-and-sleep methods, with the benefit of avoiding general anaesthesia. It also has high spatial resolution and enables simultaneous assessment of extracardiac structures such as the pulmonary parenchyma, pleura, and skeleton. The relative disadvantage is that the patient is exposed to radiation and receives an iodine contrast agent with potential risk for renal function impairment (although this risk is low when using iso-osmolar contrast agents) [4, 5].

Photon-counting CT (PCCT) was recently made available for clinical use. It yields images with negligible electronic noise and improved contrast between soft tissue and iodinated contrast agents [6]. However, this means that the images look different from conventional CT images, further dependent on the chosen reconstruction algorithm. This new CT method may potentially impact diagnostic accuracy because the observer’s experience is different.

PCCT provides higher spatial resolution than conventional CT, as well as intrinsic spectral information in every scan [7]. Thus, image quality may be improved with a lower radiation dose. A study on small children that used PCCT at 90 kV for diagnosis of congenital heart defects achieved higher image quality than conventional CT but yet reported a similar effective dose (E_eff_) [8].

It is unknown if the essentially noise-free PCCT images impact observers impression of diagnostic quality, and it is unknown which PCCT radiation dose is adequate for children with congenital heart defects. It also remains to be investigated to what extent radiation dose is reduced when moving from 90 kV to 70 kV using PCCT.

The aim of this study was therefore to compare the diagnostic quality and radiation dose in paediatric non-gated cardiovascular PCCT for diagnosis of congenital heart defects at 70 kV and 90 kV.

## Material and methods

The regional ethics committee approved this retrospective study using pseudonymised data from patients referred for a clinical examination, waiving the need for individual consent. Paediatric non-gated cardiovascular PCCT (Naeotom Alpha, Siemens Healthineers, Erlangen, Germany) examinations with intravenous contrast agent completed between 30th September 2021 and 1st March 2023 in children with suspected or confirmed congenital heart defects were eligible. Initially, only 90 kV protocols were available as this was an early installation. After 70 kV was made available by the vendor, all clinical examinations were performed at 70 kV, which also limited the number of 90 kV examinations in the current study. The aim was to include the same number of examinations using 70 kV as had been completed using 90 kV.

### Photon-counting computed tomography examination and settings

Neonates and infants were positioned in a vacuum pillow and imaged during free breathing. Small children between 1 year and 3 years were sedated with Propofol (Sandoz AS, Novartis, Stockholm, Sweden) according to clinical routine, and imaged during free breathing. Children above 3 years were generally not sedated, and images were acquired during breathhold when possible.

Tube voltage was 70 kV or 90 kV. Table 1 shows PCCT parameters. Different monoenergetic levels were tested before the current study as part of clinical optimisation, and 55 keV was considered the optimal monoenergetic level for visualisation of the cardiac chambers and the thoracic vessels. Therefore, the current study used 55 keV throughout.

**Table 1:** Photon-counting computed tomography parameters

Tube voltage	70 kV	90 kV
Automated tube current modulation	CARE dose 4-D	CARE dose 4-D
Monoenergetic level (keV)	55	55
Rotation time (s)	0.25	0.25
Slice thickness (mm)	0.6	0.6
Increment (mm)	0.4	0.4
Kernel	Bv44	Bv40, Bv44
Quantum iterative reconstruction level	4	2 (57%), 3 (17%), 4 (26%)
Acquisition	144 × 0.4	144 × 0.4
Pitch	3.2	3.2
Field of view (mm)	300	300
Image quality level	70	70

*Bv* body-vascular, *CARE* combined applications to reduce exposure, *D* dimensional

During the initial phase of clinical acquisitions, different reconstruction algorithms were tested to find the optimal quantum iterative reconstruction (QIR) level as this was the first release of the system. Furthermore, reconstruction algorithms also changed with software upgrades. This explains why different QIR levels are present for 90 kV in the current study (Table 1), whereas 70 kV protocols applied the same QIR level throughout. The clinical optimisation process for choosing QIR 4 as the superior level was by consensus discussions among all paediatric cardiovascular radiologists in the department (including those not acting as observers in the current study), in a side-by-side comparison of QIR 2, QIR 3, and QIR 4 reconstructed images.

Finally, the selected image quality level 70 means that the system’s dose modulation compensated for the lower voltage, leading to less impact of noise that would otherwise increase with lower voltage.

### Radiation exposure and contrast agent administration

The radiation dose parameters CT dose index (CTDI_vol_), dose-length product (DLP), and size-specific dose estimate (SSDE) were extracted from the PCCT system, and E_eff_ was calculated based on DLP using age-dependent conversion factors [9].

Iodixanol 270 mg I/ml (GE Healthcare, Stockholm, Sweden) was administered using a MEDRAD Centargo injector (Bayer Pharmaceuticals, Leverkusen, Germany) with 15 s bolus length. For bodyweight ≤ 10 kg, a lower extremity peripheral vein was used for contrast agent administration, and for bodyweight > 10 kg an upper extremity peripheral vein was used. Preloading of contrast agent was applied in patients with bodyweight ≤ 10 kg for the programmed injection of contrast agent to be delivered directly without intermediate saline.

For 70 kV, an optimal dilute concentration of 189 mg I/ml was determined as part of clinical optimisation, with constant injected volume and rate. For 90 kV, the clinical routine dose of 270 mg I/ml was used.

### Image analysis

Before the evaluation of study cases, the observers participated in a session with other PCCT congenital heart defect cases to obtain a common assessment basis.

All study cases were fully anonymised, including the removal of all personal identifying information, date and time of acquisition, kV level, QIR level, contrast density, and all other scan information. Also, 70 kV and 90 kV cases were randomised, although perceived image differences related to the essentially noise-free images at 70 kV could not be overcome. Four blinded paediatric cardiobascular radiologists (F.S., P.W., M.W., and S.M. with 30, 28, 24, and 20 years’ experience, respectively) independently assessed the PCCT examinations with regard to overall and specific diagnostic quality.

Overall diagnostic quality was defined as how well the examination in total could answer the clinical questions. Specific diagnostic quality was defined as how well the anatomical structures of clinical value to the individual congenital heart defect case were visualised for diagnosis. The examinations were scored 1–4 where 4 corresponded to “high diagnostic quality”, 3 “acceptable diagnostic quality”, 2 “low diagnostic quality”, and 1 “insufficient diagnostic quality”.

All examinations were evaluated as per clinical routine with four image stacks prepared for the observers’ convenience: 0.6 mm transverse images, and 2 mm images in the transverse, coronal, and sagittal planes. Multiplanar reconstruction and volume rendering based on the 0.6 mm images were used as in clinical routine.

### Statistical analysis

Statistical analyses were performed in Prism 9.5.1 (GraphPad Software, San Diego, CA) and in R [10, 11]. Values are reported as median (range) or median [interquartile range; IQR]. Due to the low score variability, standard interrater reliability measures for ordinal data are misleading. As observers were aligned in scoring before the study, chance is not the main driver for agreement. Therefore, percent rater agreement is presented. However, for comparison with other studies, intraclass correlation is also reported, assessed using a two-way, consistency, average-measures model. Mann–Whitney’s and Jonckheere-Terpstra’s tests were applied to test for score differences and for trends between E_eff_ and scores, respectively [12]. Differences between groups for 70 kV and 90 kV were assessed, and radiation dose differences were also assessed after correction for confounders. *P* < 0.05 was considered to show statistically significant differences.

## Results

**Fig. 1:**
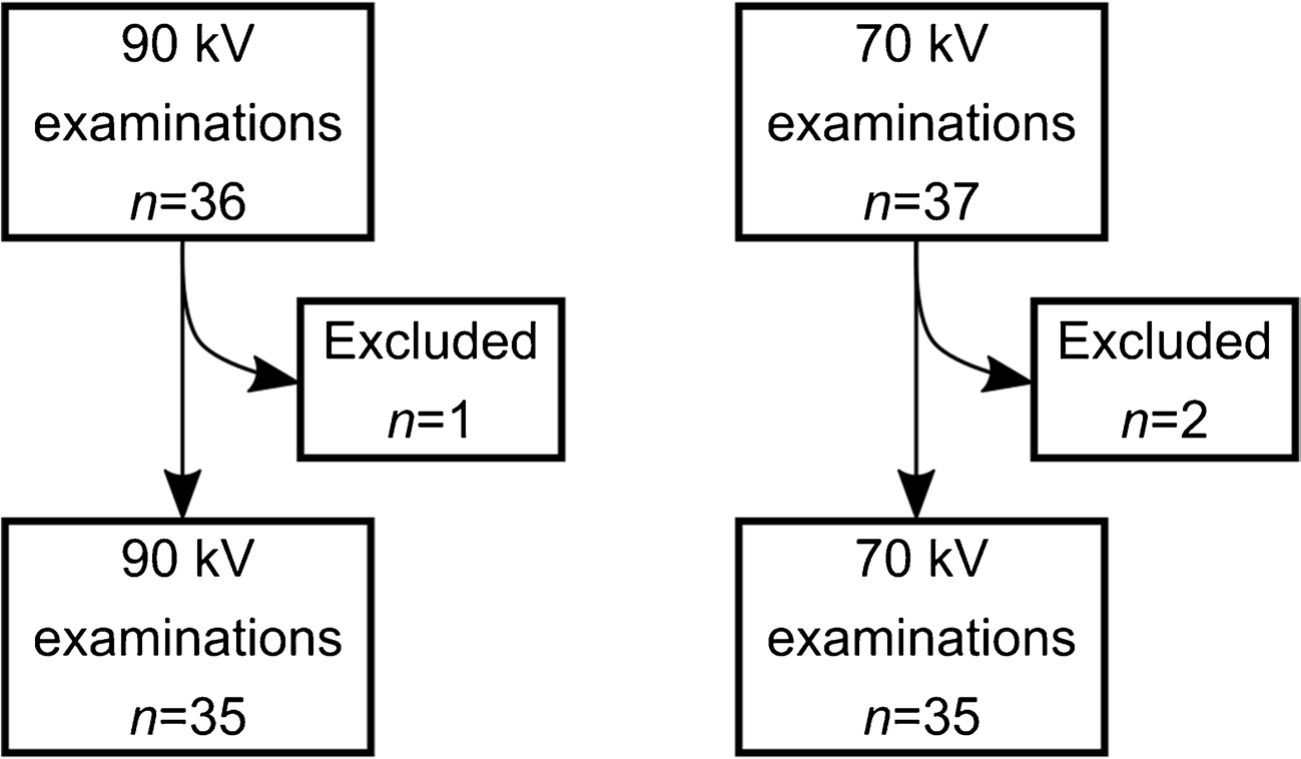
Inclusion and exclusion flowchart. As this was an early installation of the clinical photon-counting computed tomography system, only 90 kV protocols were initially available. Therefore, consecutive patients were included at 90 kV until 70 kV protocols were made available by the vendor. Thereafter, as the clinical standard was to use the then available 70 kV protocols, the aim was to include as many examinations using 70 kV as already included using 90 kV. For 90 kV, one patient was excluded due to failed contrast agent timing and no possibility to reacquire the scan on the same occasion, and for 70 kV two patients were excluded due to corrupt dose data reported by the system

Figure 1 shows the inclusion and exclusion flow chart. Table 2 shows patient characteristics including distribution of cardiovascular malformations. Although there were differences in the distribution of cardiovascular defects between the 70 kV and 90 kV groups, there was no difference between groups for age *(P* > 0.99), height (*P* = 0.86), or weight (*P* = 0.83). However, scan length differed (*P* = 0.002) between 70 kV and 90 kV groups, and therefore comparisons were made between radiation doses for 70 kV and 90 kV both using all available data and corrected for different scan lengths by matching. As expected, there was a numerical change in the *P*-value for DLP when adapting for scan length, but not for CTDI_vol_, SSDE, or E_eff_.

**Table 2:** Patient characteristics

Tube voltage	70 kV	90 kV	*P*-value
Number of patients^a^	35 (22 male)	35 (18 male)	
Age^b^	24 months (2 days–16 years)	8 months (2 days–17 years)	> 0.99
Weight (kg)^b^	12 (2–53)	8 (3–75)	0.83
Height (cm)^b^	89 (46–170)	78 (51–190)	0.86
Scan length (cm)^b^	22 (14–32)	17 (11–39)	0.002
Heart defects^a^			
Transposition of the great arteries	5	0	
Atrioventricular septal defect	1	5	
Coarctation of the aorta	5	5	
Pulmonary atresia	8	3	
Arteriopathy	0	3	
Single ventricle	8	3	
Double outlet right ventricle	1	2	
Ventricular septal defect	0	2	
Tetralogy of Fallot	2	2	
Vascular ring	0	2	
Total or partial anomalous pulmonary venous drainage	1	2	
Valvular stenosis	0	2	
Other	4	4	

^a^Number, ^b^median (range)

Table 3 shows the quality scores for all observers, with a median of 4 (high diagnostic quality) for all observers for both 70 kV and 90 kV. However, all observers scored some 90 kV cases below 3, whereas for 70 kV there were no scores below 3. Sum-of-ranks was different between 70 kV and 90 kV for observer 2 for overall diagnostic scoring (median 4 vs 4, sum-of-ranks 1,032 vs 1,454; *P* = 0.001; all other *P*-values > 0.34), and for observer 3 for specific diagnostic scoring (median 4 vs 4, sum-of-ranks 1,129 vs 1,357; *P* = 0.03; all other *P*-values > 0.06).

**Table 3:** Overall and specific diagnostic quality scores

	Overall diagnostic score^a^	Specific diagnostic score^a^
Tube voltage 70 kV		
Observer 1	4 [4–4] (3–4)	4 [4–4] (3–4)
Observer 2	4 [4–4] (3–4)	4 [4–4] (3–4)
Observer 3	4 [4–4] (3–4)	4 [4–4] (3–4)
Observer 4	4 [3–4] (3–4)	4 [3.5–4] (3–4)
Tube voltage 90 kV		
Observer 1	4 [4–4] (2–4)	4 [4–4] (2–4)
Observer 2	4 [3–4] (2–4)	4 [4–4] (2–4)
Observer 3	4 [3.75–4] (2–4)	4 [3.75–4] (2–4)
Observer 4	4 [3–4] (1–4)	4 [4–4] (1–4)

^a^Median [interquartile range] (range)

The average percent rater agreement for 70 kV was 79% (range 66–94%) for overall scores, and 89% (range 80–97%) for specific scores. For 90 kV, the average percent rater agreement for overall scores was 72% (range 60–77%) and for specific scores 86% (range 83–89%).

The intraclass correlation for 70 kV was 0.58 (95% confidence interval [CI] 0.26–0.75) for overall scores, and 0.71 (95% CI 0.51–0.84) for specific scores. For 90 kV, intraclass correlation for overall scores was 0.84 (95% CI 0.72–0.91) and for specific scores 0.85 (95% CI 0.75–0.92).

**Fig. 2:**
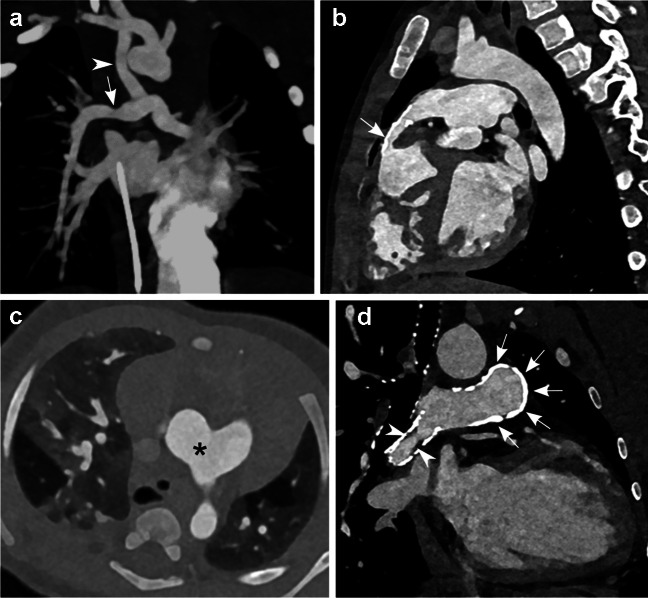
Examples of contrast-enhanced photon-counting computed tomography (PCCT) images using the 70 kV protocol. **a** Coronal maximum intensity projection with a slice thickness of 6 mm in a 14-day-old boy with pulmonary atresia, who had received a modified Blalock-Thomas-Taussig shunt and later desaturated. The image shows an open shunt (*arrowhead*) between the right subclavian and pulmonary arteries with a slight narrowing in the proximal and distal anastomosis and a stenosis (*arrow*) in the right pulmonary artery. This case had a median overall diagnostic score of 4 and a median specific diagnostic score of 4 with a size-specific dose estimate of 0.32 mGy and an effective dose of 0.15 mSv. **b** Sagittal oblique image with a slice thickness of 0.6 mm in a 5-year-old boy with tetralogy of Fallot, corrected at four months of age with a pulmonary conduit. Echocardiography showed a suspected stenosis of the pulmonary conduit, which was clearly shown using PCCT (*arrow*). This case had a median overall diagnostic score of 4 and a median specific diagnostic score of 4 with a size-specific dose estimate of 1.16 mGy and an effective dose of 0.52 mSv. **c** Transaxial image with a slice thickness of 0.6 mm in a 3-month-old boy in whom echocardiography showed a suspected aortopulmonary window and anomalous origin of pulmonary artery branches. The PCCT image shows a large aortopulmonary window between the ascending aorta and the distal part of the pulmonary trunk (*asterisk*). This case had a median overall diagnostic score of 4 and a median specific diagnostic score of 4 with a size-specific dose estimate of 0.41 mGy and an effective dose of 0.30 mSv. **d** Coronal oblique image with a slice thickness of 0.6 mm in a 16-year-old girl born with pulmonary atresia, ventricular septal defect, and major aortopulmonary collateral arteries, unifocalised to a pulmonary conduit. Multiple stents were inserted due to stenosis of the pulmonary artery branches. The PCCT image shows a heavily calcified pulmonary conduit (*arrows*) and a pulmonary artery stent with intimal proliferation (*arrowheads*). Note the good visualisation of vessel lumens despite the presence of dense calcifications and a metal stent. This case had a median overall diagnostic score of 4 and a median specific diagnostic score of 4 with a size-specific dose estimate of 1.81 mGy and an effective dose of 0.80 mSv

**Fig. 3:**
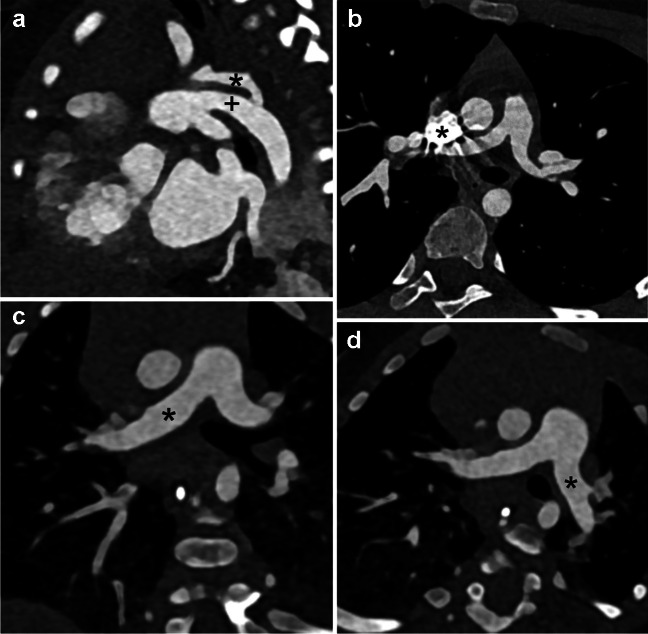
Examples of contrast-enhanced photon-counting computed tomography (PCCT) images using 90 kV protocols. **a** Sagittal oblique image with a slice thickness of 0.6 mm in an 8-day-old girl with suspected coarctation of the aorta by echocardiography. PCCT shows a hypoplastic arch (*asterisk*) with hypoplasia of the aortic isthmus and a large arterial duct (*plus sign*). This case had a median overall diagnostic score of 4 and a median specific diagnostic score of 4 with a size-specific dose estimate of 0.63 mGy and an effective dose of 0.22 mSv. **b** Transaxial image with a slice thickness of 0.6 mm in an 8-year-old girl with arterial vasculopathy, status post dilatation of the pulmonary arteries and the ascending aorta due to stenosis. Note the streak artefact from the relatively dense contrast medium (*asterisk*). Despite this, the pulmonary arteries and the ascending aorta were sufficiently visualised for diagnostic purposes. This case had a median overall diagnostic score of 3 and a median specific diagnostic score of 4 with a size-specific dose estimate of 1.09 mGy and an effective dose of 0.24 mSv. **c**, **d** Coronal (**c**) and transaxial (**d**) oblique images with a slice thickness of 0.6 mm depict the right (**c**; *asterisk*), and left (**d**; *asterisk*) pulmonary arteries in a 5-month-old prematurely born girl with idiopathic pulmonary arterial hypertension. The pulmonary arteries were well visualised without stenosis. This case had a median overall diagnostic score of 3 and a median specific diagnostic score of 3 with an effective dose of 0.30 mSv

Figures 2 and 3 show clinical examples of 70 kV and 90 kV examinations. Maximum intensity projections or 0.6 mm images are presented to more clearly show relevant anatomical structures.

**Fig. 4:**
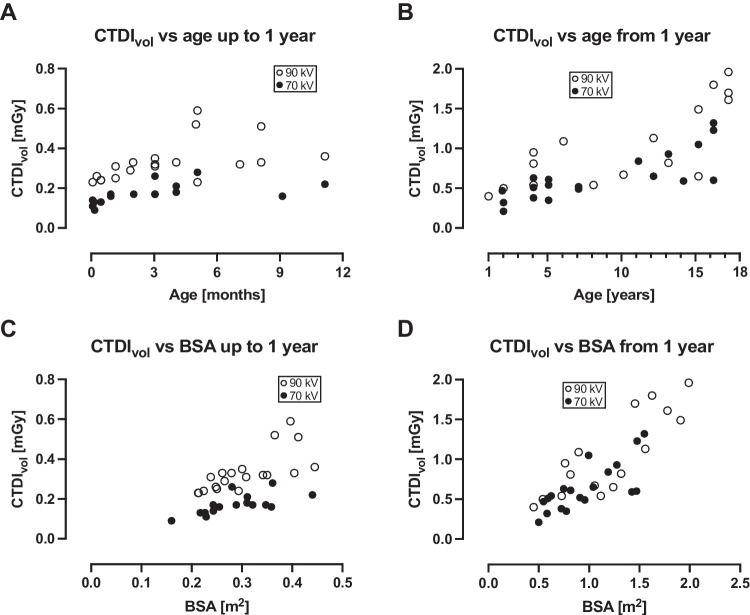
Computed tomography dose index (CTDI_vol_) versus age and body surface area (BSA). For all patients below 1 year of age, CTDI_vol_ was less than 0.3 mGy for 70 kV, and less than 0.6 mGy for 90 kV. For patients between 1 year and 17 years of age, CTDI_vol_ increased with age for both 70 kV and 90 kV. It also increased with BSA for both 70 kV and 90 kV, but to a larger degree for 90 kV

**Fig. 5:**
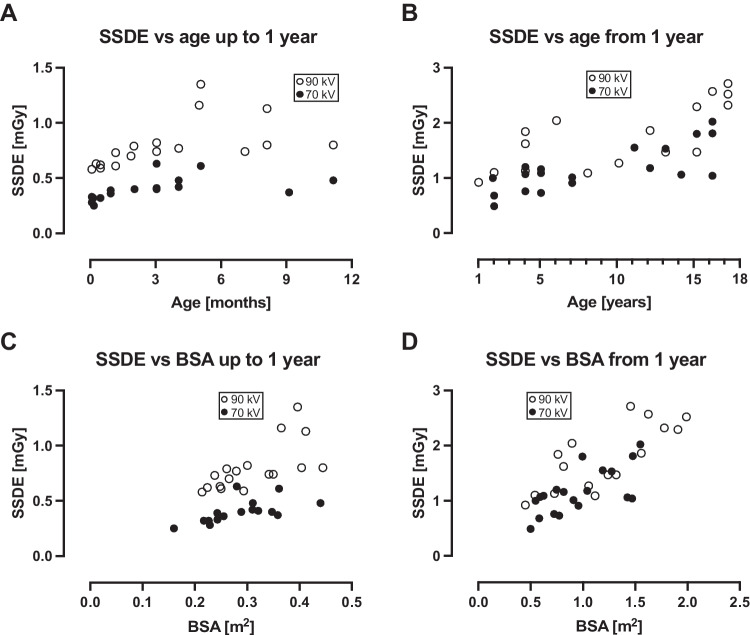
Size-specific dose estimate (SSDE) versus age and body surface area (BSA). For all patients below 1 year of age, SSDE was less than 0.65 mGy for 70 kV, and less than 1.4 mGy for 90 kV. For patients between 1 year and 17 years of age, SSDE was less than 2.05 mGy for 70 kV and less than 2.75 mGy for 90 kV. SSDE increased with BSA for both 70 kV and 90 kV, but to a larger degree for 90 kV

**Fig. 6:**
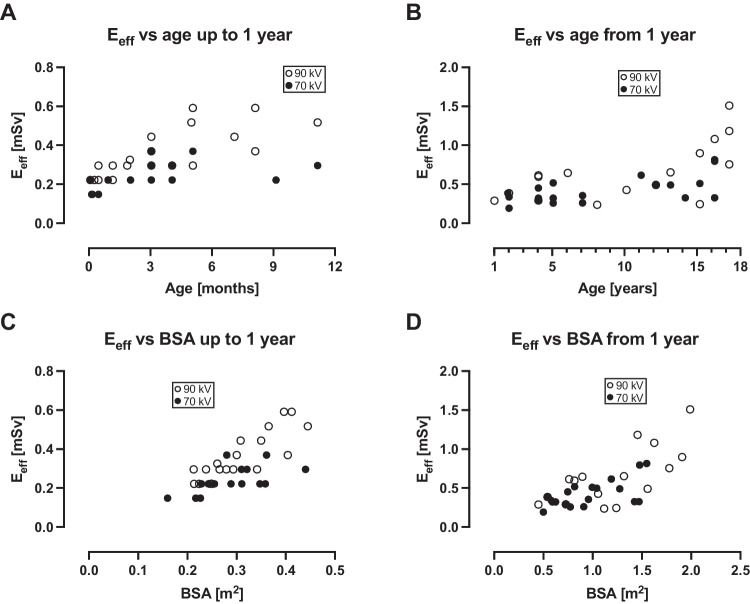
Effective dose (E_eff_) versus age and body surface area (BSA). For most patients below 1 year of age, E_eff_ was less than 0.5 mSv for 70kV, and less than 1 mSv for all patients between 1 year and 17 years of age. E_eff_ increased with BSA for both 70 kV and 90 kV, but to a larger degree for 90 kV

Radiation dose parameters are shown in Table 4, and radiation dose parameters versus age and body surface area in Fig. 4 for CTDI_vol_, in Fig. 5 for SSDE, and in Fig. 6 for E_eff_.

**Table 4:** Radiation dose parameters at 70 kV and 90 kV

	70 kV	90 kV	*P*-value
CTDI_vol_ (mGy)			
All patients 0–17 years	0.32 (0.09–1.32)	0.50 (0.23–1.96)	0.01
0–12 months	0.17 (0.09–0.28)	0.32 (0.23–0.59)	< 0.0001
SSDE (mGy)			
All patients 0–17 years	0.68 (0.25–2.02)	1.1 (0.58–2.71)	< 0.001
0–12 months	0.40 (0.25–0.63)	0.74 (0.58–1.35)	< 0.0001
E_eff_ (mSv)			
All patients 0–17 years	0.30 (0.15–0.82)	0.39 (0.22–1.51)	0.01
0–12 months	0.22 (0.15–0.37)	0.30 (0.22–0.59)	0.001
DLP (mGy × cm) not matched for scan length			
All patients 0–17 years	7 (2–40)	7 (3–74)	0.22
0–12 months	3 (2–5)	5.5 (4–8)	< 0.0001
DLP (mGy × cm) matched for scan length			
All patients 0–17 years	5 (2–40)	8 (4–53)	0.02
0–12 months	3 (2–5)	4 (3–8)	0.001

*CTDI*_*vol*_ computed tomography dose index, *DLP* dose-length product, *E*_*eff*_ effective dose, *SSDE* size-specific dose estimate. Values are reported for all patients, and for the smallest and most radiosensitive patients, i.e. those aged 0–12 months

Jonckheere-Terpstra’s tests showed that there was no trend between E_eff_ and overall scores (70 kV: *P* = 0.55; 90 kV: *P* = 0.98) or between E_eff_ and specific scores (70 kV: *P* = 0.55; 90 kV: *P* = 0.29).

## Discussion

This study shows that PCCT yields images of high diagnostic quality for paediatric congenital heart defect examinations with a concomitant low radiation dose. Further, vessels were well-defined with a diluted contrast agent dose at 70 kV, with scoring equal to or higher than the full contrast dose used for 90 kV.

Paediatric cardiovascular PCCT therefore has the potential to become the primary complementary modality to echocardiography in congenital heart defects due to its high diagnostic quality, low radiation dose, and low contrast agent dose. This is particularly important in complex congenital heart defects where repeat examinations are needed in small radiosensitive children.

Although magnetic resonance imaging has the benefit of providing both functional and anatomical information, it has limited spatial resolution. CT will thus remain the gold standard for anatomical assessment of small vessels in conditions such as congenital heart defects with pulmonary atresia, pulmonary vein stenosis, collaterals, or coronary artery anomalies, where precise delineation of anatomy is central to planning and guiding surgical or interventional treatment.

All cases were of high diagnostic quality and could answer the clinical questions. Observers gave scores of 3 and 4 to the 70 kV images, but scores from 1 to 4 for the 90 kV images. This indicates that the essentially noise-free images at 70 kV do not impact observers negatively. Also, agreement between observers was generally high. In regard to image quality, this is similar to a 90 kV PCCT study showing a 97% success rate; however, that study only showed moderate or poor interreader agreement [8].

The current study showed a median E_eff_ of 0.39 mSv at 90 kV for all patients between birth and 17 years. For those below 1 year of age, median E_eff_ was 0.30 mSv. Both values are lower than those in the only previous comparable PCCT study, where mean E_eff_ was 0.50 mSv (± 0.23 mSv) at 90 kV, even though the children in that study (66 [10–161] days) were much younger than in the current study [8]. Further, Dirrichs et al. [8] showed a mean DLP at 90 kV of 14.3 (± 6.6; range 3–42) mGy × cm for their very young children, compared with the current study’s mean DLP of 4 (range 3–8) mGy × cm. It is unclear why the previous study showed such high radiation exposure, but possible explanations include larger scan length or the use of a lead apron which may increase radiation dose unless automated tube current modulation is turned off [13].

For 70 kV, the current study showed a median E_eff_ of 0.30 mSv with all ages up to 17 years included, and for those below 1 year of age, the median E_eff_ was 0.22 mSv. In comparison, a recent 70 kV dual-source conventional CT study that only included young children (3.5 [0.2–6.6] months) showed a median E_eff_ of 0.20 mSv [14].

Different CT methods, dose calculations, and use of weighting factors make direct comparison between studies challenging. There is nevertheless a trend towards reduced radiation doses for non-gated cardiovascular CT, where the current PCCT study shows a further dose reduction with E_eff_ of 0.15–0.82 mSv for 70 kV and 0.22–1.51 mSv for 90 kV in children between 0 and 17 years. This is lower than previous conventional non-gated CT studies in small children between 0 years and 4.5 years, where E_eff_ was between 0.20 and 1.95 mSv [15–17].

Both CTDI_vol_ and DLP are commonly used to estimate radiation exposure. They are however relatively blunt measures—especially DLP—when comparing radiation doses, which is supported by the current results. For comparisons between studies, SSDE may be preferred as a reliable tool to estimate average radiation dose depending both on CT parameters and size of the specific patient, even though it does not include organ or tissue weighting factors [18]. So far, relatively few studies have presented SSDE, and none of them have used PCCT for congenital heart defects. The current study, by presenting SSDE, provides an opportunity for future comparisons.

This study has limitations. The PCCT software was updated during the study. However, image scores did not change with time or stepwise after software updates, and so the impact of the updates is considered small. Even though diagnostic quality was assessed it would be appropriate for clinical impact to study anatomy by PCCT, and compare it to anatomy during surgery. Surgery was not performed in all patients and there is no other examination more accurate than PCCT available for comparison. Therefore, neither accuracy nor potentially missed diagnoses were assessed. It is however unlikely that accuracy is decreased using PCCT compared to conventional CT either for congenital heart defects as studied here, or for other possible associated cardiovascular abnormalities. Finally, there is no international consensus regarding which QIR level to use for congenital heart defects, but QIR 4 in its present form as used in the current study is now preferred in several centres.

## Conclusion

Paediatric cardiovascular PCCT yields images for congenital heart defects of high diagnostic quality with low radiation dose at both 70 kV and 90 kV.

## Data Availability

Data supporting the findings of this study are not openly available due to reasons of sensitivity. Data are located in controlled access data storage at Skåne University Hospital. Data may be made available upon reasonable request to the corresponding author.
